# Mapping Power Law Distributions in Digital Health Social Networks: Methods, Interpretations, and Practical Implications

**DOI:** 10.2196/jmir.4297

**Published:** 2015-06-25

**Authors:** Trevor van Mierlo, Douglas Hyatt, Andrew T Ching

**Affiliations:** ^1^ Research Associate, Henley Business School, University of Reading Oxfordshire United Kingdom; ^2^ Evolution Health Systems Inc Toronto, ON Canada; ^3^ Rotman School of Managment University of Toronto Toronto, ON Canada

**Keywords:** social networks, eHealth, 1% rule, Pareto Principal, power law, 90-9-1 principle, moderated support, peer-to-peer support

## Abstract

**Background:**

Social networks are common in digital health. A new stream of research is beginning to investigate the mechanisms of digital health social networks (DHSNs), how they are structured, how they function, and how their growth can be nurtured and managed. DHSNs increase in value when additional content is added, and the structure of networks may resemble the characteristics of power laws. Power laws are contrary to traditional Gaussian averages in that they demonstrate correlated phenomena.

**Objectives:**

The objective of this study is to investigate whether the distribution frequency in four DHSNs can be characterized as following a power law. A second objective is to describe the method used to determine the comparison.

**Methods:**

Data from four DHSNs—Alcohol Help Center (AHC), Depression Center (DC), Panic Center (PC), and Stop Smoking Center (SSC)—were compared to power law distributions. To assist future researchers and managers, the 5-step methodology used to analyze and compare datasets is described.

**Results:**

All four DHSNs were found to have right-skewed distributions, indicating the data were not normally distributed. When power trend lines were added to each frequency distribution, *R* ^2^ values indicated that, to a very high degree, the variance in post frequencies can be explained by actor rank (AHC .962, DC .975, PC .969, SSC .95). Spearman correlations provided further indication of the strength and statistical significance of the relationship (AHC .987. DC .967, PC .983, SSC .993, *P*<.001).

**Conclusions:**

This is the first study to investigate power distributions across multiple DHSNs, each addressing a unique condition. Results indicate that despite vast differences in theme, content, and length of existence, DHSNs follow properties of power laws. The structure of DHSNs is important as it gives insight to researchers and managers into the nature and mechanisms of network functionality. The 5-step process undertaken to compare actor contribution patterns can be replicated in networks that are managed by other organizations, and we conjecture that patterns observed in this study could be found in other DHSNs. Future research should analyze network growth over time and examine the characteristics and survival rates of superusers.

## Introduction

### Background

Empirical examination of digital health social networks (DHSNs) began in the mid-1980s. In 1986, Schneider examined abstinence rates from smoking among 28 actors of an online system named the Electronic Information Exchange System (EIES) [1]. Actors logged on to EIES by typing the word “smoker” and could read and post messages to a bulletin board. During the same year, Robinson and Walters outlined Health-Net, an interactive computer network linking personal computers in student residences, libraries, academic buildings, and the Student Health Center at Stanford University [2]. Like EIES, Health-Net contained a bulletin board. These researchers all noted the potential impact of these networks on personal health, especially in regards to information access and knowledge sharing.

Decades later, DHSNs, otherwise known as bulletin boards, peer-to-peer support groups, online forums, or computer-mediated communication now proliferate the digital health landscape. As of December 2014, over 40,000 health-related communities exist on Yahoo! Groups. PatientsLikeMe, a for-profit health care company focusing on peer-to-peer support, has communities for over 2300 conditions. In 2013, Bender et al identified and examined 111 DHSNs dedicated to breast cancer survivors, with extensive archives of personal experiences [3].

The Internet also continues to evolve as an important health resource. A 2013 Pew Research Center report found that within the past year 59% of US adults used the Internet to search for health information, and 26% of Internet users read or watched someone else’s experience about a health or medical issue [4].

Although the research community is in the process of establishing the efficacy of DHSNs [5], peer-to-peer support groups remain an important component of the digital health ecosystem. A separate stream of research is evolving, which seeks to understand the mechanisms of DHSNs, how they are structured, how they function, and how their growth can be nurtured and managed. Other disciplines have analyzed complex networks, and measured specific interactions within [6], yet their theories and models have yet to be rigorously applied to digital health.

### Network Effects

For decades, the fields of economics and marketing have sought to understand the structure, stagnation, growth, and distribution patterns of networks. The study of networks in demand-side economics has found that the value of a product or service is directly related to the number of others who use it [7-10]. This increase in value, otherwise known as a *positive network externality*, occurs with each sale of an additional unit.

This increase in value can be illustrated in many consumer goods. An example is FaceTime, a popular feature of Apple products. FaceTime is a videotelephony service (or video call app) that allows consumers to talk with each other via Voice over Internet Protocol (VoIP). FaceTime is available only on Apple products, so consumers must purchase an Apple product in order to join the FaceTime network. There were an estimated 19 million FaceTime-equipped devices in October 2010, growing to over 300 million by the end of 2012 [11]. For consumers (and presumably Apple), the value of the FaceTime network continues to increase with the sale of each additional Apple device.

### Power Laws and Power Curves

A power law is an exponential relationship between two values that is scaled and is proportional. A power curve is the graphical representation of this phenomenon.

If plotted on a graph, the distinguishing feature of a power curve is a straight line with a slope of b or an equation of y=-x+b; the closer the data fit the straight line, the greater chance of the graphed relationship being defined as a power curve.

An example of a well-known power law is the Pareto Principal, colloquially known as the 80-20 rule. In the late 19th century, the Italian scholar Vilfredo Pareto noted that 80% of the land in Italy was owned by 20% of the population [12]. Likewise, it is common for those in business to note that 80% of their sales are generated from 20% of their customers or that 80% of absences can be attributed to 20% of staff.

One specific type of power law is a Zipfian distribution, otherwise known as Zipf’s law, eponymously named after George Kingsley Zipf, an American linguist and philologist who was a university lecturer at Harvard University [13]. Zipf first noted a statistical relationship in the frequency of word use but extended his method to other subjects, such as the size of cities and concentration of economic power [14].

Examples of power laws are ubiquitous. One resource lists over 80 types of natural and social power law phenomena in fields such as physics (eg, brush-fire damage, water levels in the Nile, earthquakes, size of asteroid hits), biology (eg, genetic circuitry, tumor growth, death from heart attack, predicting premature births, mass extinctions), social science (eg, word use, structure of World Wide Web, publications and citations, global terrorism events, traffic jams), and management research (eg, cotton prices, distribution of wealth, intra-firm decision events, alliance networks among biotech firms) [15]. In a separate study, power law distributions were consistent in 17 of 24 datasets ranging from linguistics (count of word use), biology (protein interaction degree), ornithology (bird species sightings), meteorology (solar flare intensity), and political science (intensity of wars) [16].

Power laws have been studied extensively, with Paul Kruman, a Nobel Prize winning economist, describing the phenomenon as “disturbing” or “baffling” [17]. However, defining power relationships are important as the models can help us with intuition and to begin to understand relationships between two distinct variables.

### Distribution Patterns and Digital Health Social Networks

All networks have the potential to increase in value when an additional user or actor is added. Generally, if a network contains *n* users, potential connections between users is *n*(*n*-1). However, value creation differs among various network types.

**Figure 1:**
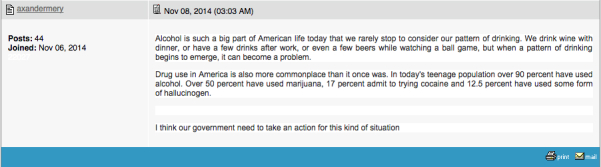
Post by actor axandermery on the social network Alcohol Help Center.

In our previous FaceTime example, network connections are ephemeral; a conversation between two actors terminates when a party ends the conversation. DHSNs differ from traditional networks as actor contributions are permanent. An actor’s post remains on the network and can be accessed or read numerous times (Figure 1).

As actor posts are permanent in DHSNs, positive network externalities occur in two instances. The first is when a new actor joins the network and creates one or more posts. The second is when an existing actor authors a new post. In both instances, the network increases in size and value is added. Not surprisingly, actors post in varying frequencies, and some actors create more posts than others. The mathematical relationship between these two quantities (number of actors and number of posts) often constitutes a power law. Power laws are in contrast with traditional Gaussian averages in that they demonstrate correlated phenomena [15].

Recent research has started to investigate the power law phenomenon in DHSNs. A 2014 study found that the 1% rule, a marketing “rule of thumb”, was consistent across four separate DHSNs [18]. Shortly afterwards, the 1% study was replicated within an Australian DHSN for depression [19]. This study confirmed the 1% rule and found that the ranked distribution of actor contributions fit a specific power law known as a Zipfian distribution.

As outlined previously, DHSNs have the potential to positively impact patients and may play a key role in normalizing disease and influencing medication and treatment adherence [4,5]. If they follow properties of power laws, managers and researchers may be able to account for, and anticipate, fluctuations in growth.

### Objective

The objective of this study was to investigate whether the distribution frequency of four DHSNs, each addressing a unique condition, could be described as power curves. To assist future researchers with assessing the distribution frequencies of other DHSNs, a second objective was to describe the method used to determine the comparison.

## Methods

### Overview

The four DHSNs used in this study are Alcohol Help Center (AHC), Depression Center (DC), Panic Center (PC), and Stop Smoking Center (SSC). All DHSNs are moderated, are free to participants, do not offer any advertising or product promotion, and are components of each website’s behavior-change program.

The DHSNs have been in existence for a considerable amount of time, ranging from 4.0 to 10.9 years (see Table 1).

**Table 1:** Digital health social networks.

	Problem drinking	Depression	Panic disorder	Smoking cessation
Date of first post	July 25, 2008	April 5, 2003	January 7, 2002	September 17, 2001
Date of last post	August 7, 2012	August 5, 2012	August 7, 2012	August 7, 2012
Days, n	1474	3411	3866	3978
Years, n	4.0	9.3	10.6	10.9
Posts, n	7148	12,583	45,032	513,586
Registrations, n	2584	5151	11,372	44,870
Actors who made at least one post, n (%)	449 (17.7%)	1230 (23.9%)	2767 (24.3%)	7963 (17.7%)

Each program has been extensively studied in the literature [20-32], and program features and functionality have been described elsewhere [18]. The major theoretical underpinnings used to develop the interventions are described in Table 2 [33-42].

**Table 2:** Theoretical underpinnings of behavior-change programs.

	Problem drinking	Depression	Panic disorder	Smoking cessation
Brief Intervention [33]	✔	✔	✔	✔
Cognitive Behavioral Therapy [34]		✔	✔	
Gamification [35]	✔	✔	✔	✔
Health Belief Model [36]	✔	✔	✔	✔
Motivational Interviewing [37]	✔	✔	✔	✔
Social Cognitive Theory [38,39]	✔	✔	✔	✔
Structured Relapse Prevention [40]	✔			✔
Targeting and Tailoring [41]	✔	✔	✔	✔
Transtheoretical Model / Stages of Change [42]	✔			✔

The four DHSNs are funded and managed by Evolution Health System Inc (EHS) and are part of the firm’s social business model. EHS is a private, research-based organization that builds evidence-based digital programs designed to increase medication and treatment adherence.

All data collection procedures adhered to international privacy guidelines [43-45] and were in accordance with the Helsinki Declaration of 1975, as revised in 2008 [46]. The study was consistent with the University Research Ethics Committee procedures at Henley Business School, University of Reading, and was exempt from full review.

A 5-step process was undertaken to compare actor contributory patterns of the four DHSNs to power curves, as follows.

### Step One

Data on all actors who posted one or more posts were imported from each DHSN’s structured query language (SQL) server database to Microsoft Excel. Actors were then ranked, with the actor creating the greatest number of posts assuming the first position, the actor creating the second greatest number of posts in the second position, and so on.

**Figure 2:**
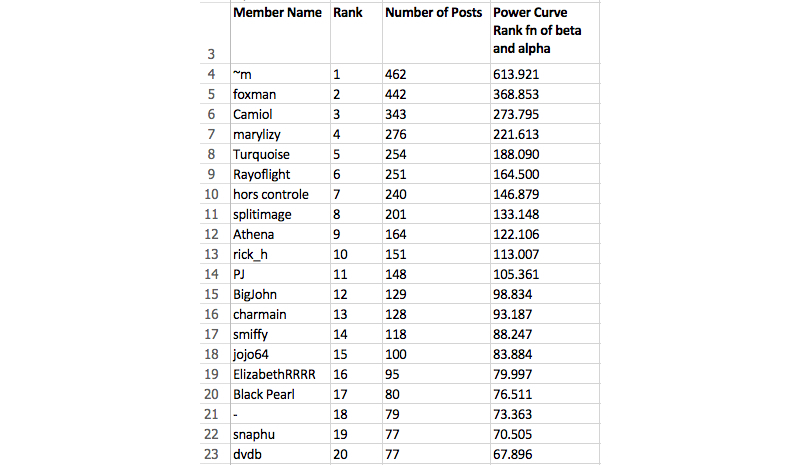
Ranking of top 20 actors contributing to Alcohol Help Center.

Figure 2 illustrates this ranking process with actors from the AHC DHSN. The actor *~m* created the greatest number of posts in the network (n=462), assuming the rank of one. This is followed by the actor *foxman* who assumed the rank of two (n=442), and the actor *Camiol*, who assumed the third rank (n=343).

### Step Two

**Figure 3:**
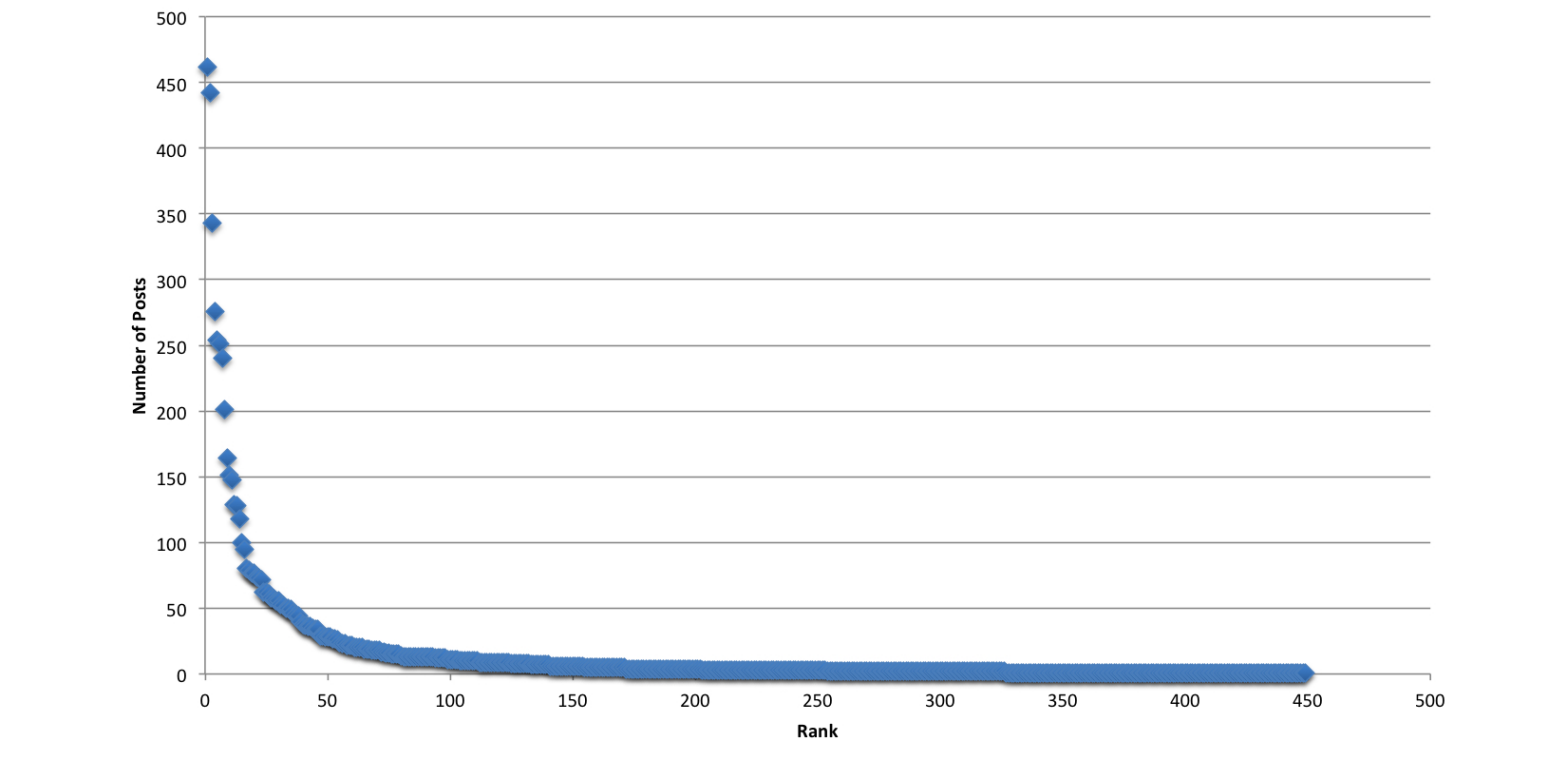
Cumulative posting trends in Alcohol Help Center.

Power distributions in ranked data have skewed distributions [14]. To visually determine the skewness, or asymmetry of the DHSNs rank distribution, X-Y scatter plots were created in Microsoft Excel (Figure 3).

### Step Three

In Excel, each actor’s ranking was mapped to an equal position on a power curve with a slope of beta defined as y=10^(alpha+beta*Log^10^x) (see Figure 2, Column D). We estimated alpha and beta for each of the networks by minimizing the sum of squared residuals based on the observed y and the predicted y.

### Step Four

**Figure 4:**
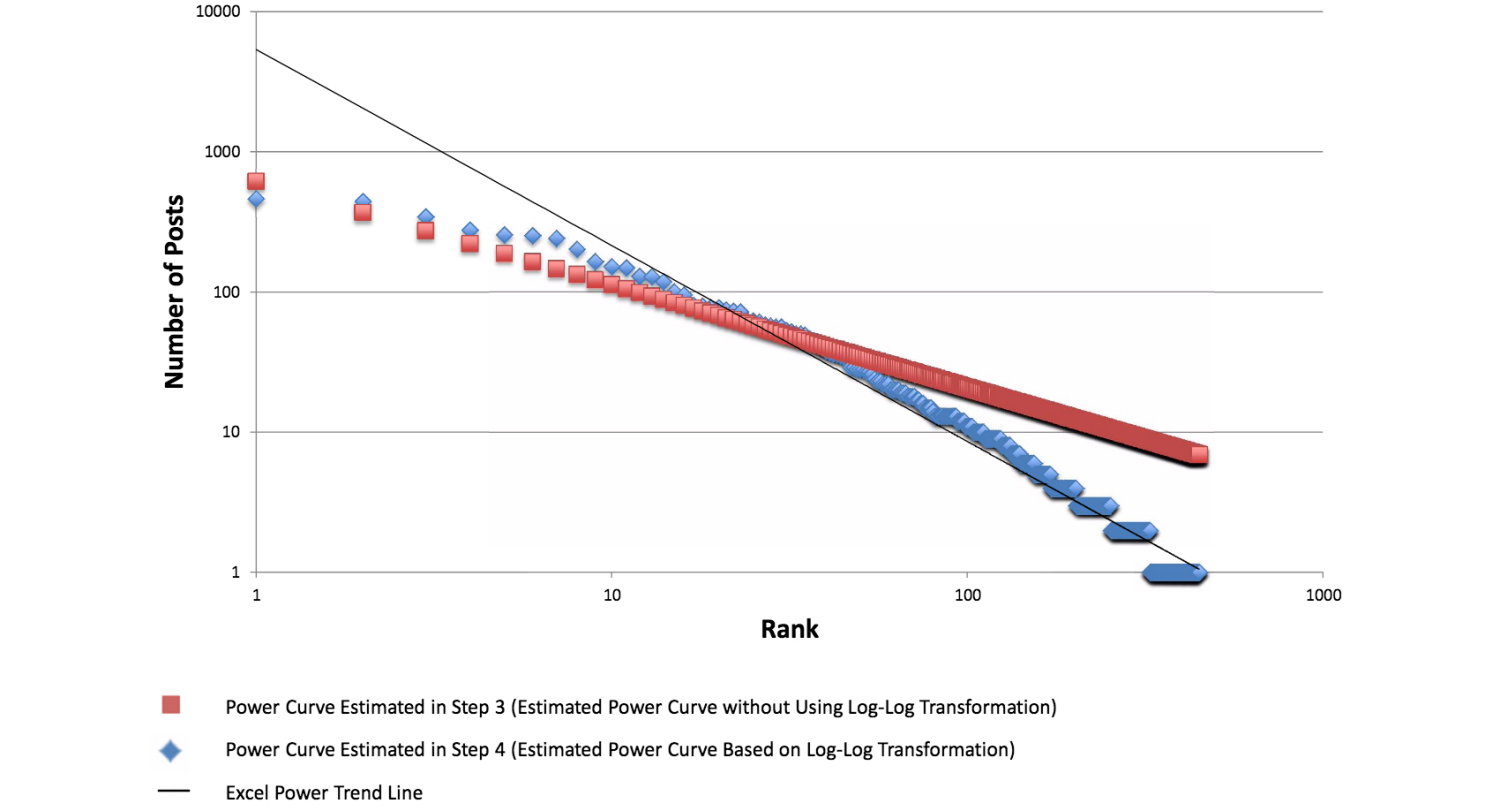
Alcohol Help Center actor ranking and power curve raking with trendline and R2 value.

To visually compare each DHSN posts with its corresponding power curve rank function of alpha and beta, X-Y scatter plots were generated in Microsoft Excel, with both axes transformed to logarithmic scales. For DHSN posts, an Excel power trend line was added with the *R* ^2^ option selected (Figure 4). In this Excel built-in option, Excel applies ordinary least squares (OLS) to estimate Log(y)=alpha + beta*Log(x) + ε. In other words, Excel estimates alpha and beta by minimizing the sum of square residuals based on the observed Log(y) and Log(predicted y). However, in Step 3, we use y and predicted y to compute sum of squared residuals. This is why the predicted line generated by Excel power trend line option in Step 4 differs from the one we generate in Step 3.

### Step Five

In SPSS, Spearman correlations were used to compare DHSN posts to the power curve rank function of alpha and beta (Columns C and D in Figure 2). Spearman correlation was employed as the comparison method because it is commonly used in non-linear distributed data and does not make assumptions about the frequency distribution of variables [47].

## Results

**Figure 5:**
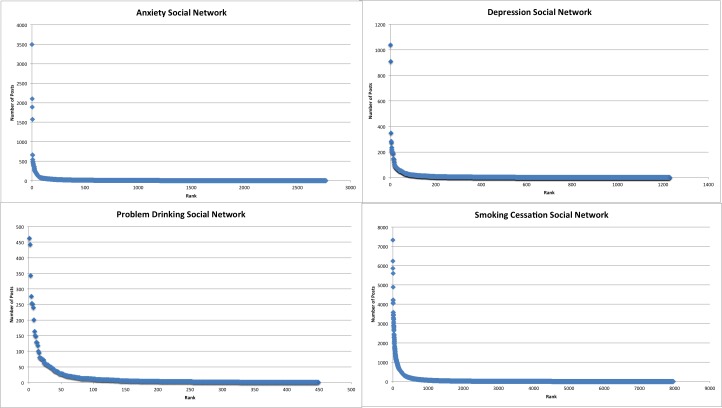
Right skewed distributions in four DHSNs.

All four DHSNs were found to have right skewed distributions, indicating that the data were not normally distributed. This also confirmed that a small number of actors created the vast majority of content (Figure 5).

**Figure 6:**
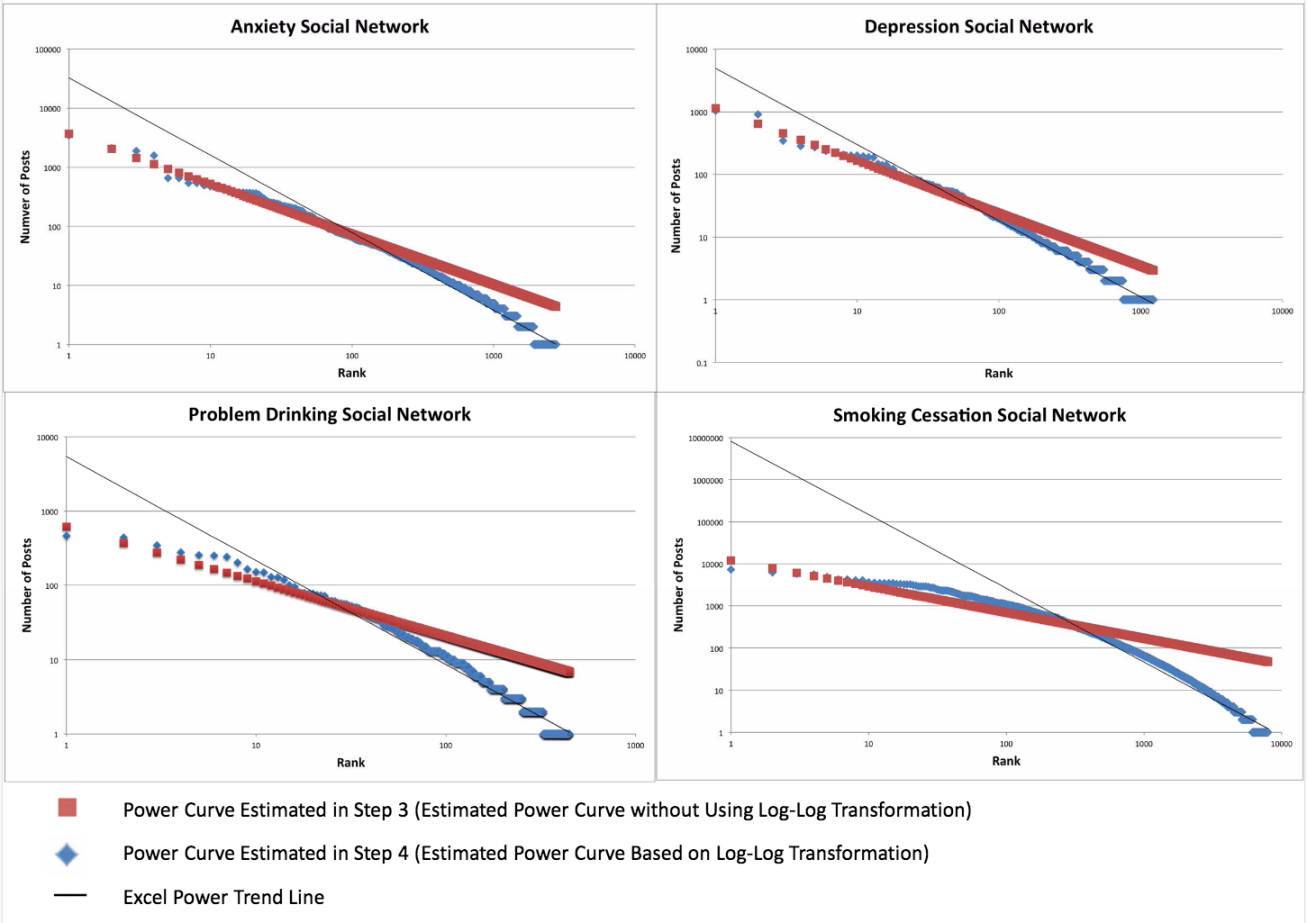
DHSN actor ranking and power curve ranking with trendline and R2 value.

When logged, each of the DHSN’s rank and post frequency data closely resembled power distributions. When Excel power trend lines were added, *R* ^2^ values indicated that to a very high degree, the variance in post frequencies is explained by actor rank (Figure 6).

To assess strength of the linear relationship between actor rank and number of contributions, and power curve rank, Spearmen correlations were calculated (Table 3).

**Table 3:** Comparison of log-log scatter plots to power curves.

Social network	*R* ^2^	Spearman correlation (sig)
Problem drinking	.96207	.987 (*P*<.001)
Anxiety	.96875	.972 (*P*<.001)
Depression	.97508	.967 (*P*<.001)
Smoking cessation	.94979	.993 (*P*<.001)

## Discussion

### Principal Findings

The four DHSNs analyzed in this experiment differed in several areas. First, they addressed unique topics (two mental health, two addictions). Second, all four groups existed for different periods of time (minimum=4.0 years, maximum=10.9), had varying actor populations (minimum=449, maximum=7963), and total number of posts (minimum=7148, maximum=513,586).

Despite differences in condition addressed, program duration and data collection period, populations, and number of post sizes, results indicate that to a high degree, the distribution patterns of DHSNs resemble that of a power law. As power laws demonstrate correlated phenomena, they can help describe the topology of DHSNs.

### Practical Implications

The results of this study have several practical implications for DHSN owners and managers. Unlike the earlier FaceTime example where actor conversations are ephemeral, all DHSN posts remain on the network, and each additional post increases network value. By following the 5-step process outlined in this manuscript, managers can map the growth of their networks and graphically isolate specific types of actors.

As positive network externalities occur with the addition of each post, it is imperative for managers to develop methods designed to retain actors who frequently create content. Past research has identified these actors as superusers [48,49]; however, very little is known about superuser demographic or psychographic characteristics.

Many DHSNs are managed by trained moderators. In health care settings, moderators are often required to read and approve posts, answer usability questions, and manage disputes. Support group moderation is a relatively new but growing profession [50], and to date there are few best practices designed to estimate labor costs. As staffing is often dependent on network size, observing power law distributions may help managers establish budgets and expenditures, such as employee recruitment or training.

### Strengths and Limitations

A strength of this study is the use of four separate DHSNs with varying topics, population sizes, and periods of existence. A second strength is that the programs are not actively advertised or promoted, and there is no cost to join or participate. This has resulted in a dataset that contains a naturalistic, self-seeking population with limited participatory barriers.

However, this same strength may also be a weakness. Typically, networks have barriers to entry such as registration fees or membership requirements. A further weakness is that all four networks are managed and maintained by the same organization, and the information architecture of the programs is similar.

Another factor to be considered is that other phenomenon may be better suited to explain network patterns. For example, many smokers make an attempt to quit at the start of a new year [51], and seasonality may be better suited to explain both short- and long-term DHSN growth.

Also deserving of consideration is that the definition of network value in this manuscript is derived from the economics literature, where the addition of each post creates a positive network externality. In practice, all posts are not of equal value. Certain posts will be frequently visited and commented on more than others, and the value of these posts are arguably greater than posts that are less popular. Trained moderators also viewed, approved, and in some cases edited all posts in this study. Some posts were also deleted due to inappropriate content. In this context, future research may refine the definition of network value.

Finally, the efficaciousness of DHSNs has yet to be firmly established in the literature. Research continues to focus on possible relationships between social network use and increased treatment adherence and measurable health outcomes.

### Future Research

To further validate our results, the method used in this study should be replicated in networks that are managed by other organizations, and it would be helpful to focus on a variety of conditions.

Future research should also analyze network growth over time through analyzing longitudinal or panel data. The 5-step method outlined in this paper could be applied to an investigation observing the strength and consistency of power distributions throughout the life span of a single DHSN.

The results of this study indicate that superusers may be important for network growth. Future research should investigate the direction of the causal relationship between superusers and network size. Future research should also seek to gain a better understanding of superuser characteristics, demographics, psychographics, and their survival rates.

Due to the availability of big data, other disciplines are now investigating the importance of the small number of consumers who account for a large percentage of profits [52,53], and health care should follow suit. Leveraging the expertise, wisdom, and experience of patients who are dedicated to sharing their knowledge and experience could possibly translate to increased treatment adherence and efficacy.

### Conclusions

This is the first study to investigate power curves across multiple DHSN. To a high degree, the rank and post frequencies of the four DHSNs hold properties of power laws. The implications of the results are important as they give insight to both researchers and managers into the nature and inner mechanisms of DHSNs. Future research examining the characteristics, survival rates, and role of superusers is required.

## Abbreviations

AHC: Alcohol Help Center
DC: Depression Center
DHSN: digital health social networks
EIES: Electronic Information Exchange System
EHS: Evolution Health Systems Inc
PC: Panic Center
SCC: Stop Smoking Center
VoIP: Voice over Internet protocol
